# Comparison of static and rolling logistic regression models on predicting invasive mechanical ventilation or death from COVID‐19—A retrospective, multicentre study

**DOI:** 10.1111/crj.13560

**Published:** 2022-11-21

**Authors:** Milo Engoren, Carlo Pancaro, Nicholas S. Yeldo, Lotfi S. Kerzabi, Nicholas Douville

**Affiliations:** ^1^ Department of Anesthesiology University of Michigan Ann Arbor Michigan USA; ^2^ Department of Anesthesiology Henry Ford Medical Center Detroit Michigan USA; ^3^ Computational Medicine and Bioinformatics University of Michigan Ann Arbor Michigan USA

**Keywords:** COVID, death, clinical decision support, clinical prediction models, logistic regression, mechanical ventilation

## Abstract

**Introduction:**

COVID‐19 virus has undergone mutations, and the introduction of vaccines and effective treatments have changed its clinical severity. We hypothesized that models that evolve may better predict invasive mechanical ventilation or death than do static models.

**Methods:**

This retrospective study of adult patients with COVID‐19 from six Michigan hospitals analysed 20 demographic, comorbid, vital sign and laboratory factors, one derived factor and nine factors representing changes in vital signs or laboratory values with time for their ability to predict death or invasive mechanical ventilation within the next 4, 8 or 24 h. Static logistic regression was constructed on the initial 300 patients and tested on the remaining 6741 patients. Rolling logistic regression was similarly constructed on the initial 300 patients, but then new patients were added, and older patients removed. Each new construction model was subsequently tested on the next patient. Static and rolling models were compared with receiver operator characteristic and precision‐recall curves.

**Results:**

Of the 7041 patients, 534 (7.6%) required invasive mechanical ventilation or died within 14 days of arrival. Rolling models improved discrimination (0.865 ± 0.010, 0.856 ± 0.007 and 0.843 ± 0.005 for the 4, 8 and 24‐h models, respectively; all *p* < 0.001 compared with the static logistic regressions with 0.827 ± 0.011, 0.794 ± 0.012 and 0.735 ± 0.012, respectively). Similarly, the areas under the precision‐recall curves improved from 0.006, 0.010 and 0.021 with the static models to 0.030, 0.045 and 0.076 for the 4‐, 8‐ and 24‐h rolling models, respectively, all *p* < 0.001.

**Conclusion:**

Rolling models with contemporaneous data maintained better metrics of performance than static models, which used older data.

## Graphical abstract

When a disease or treatment is rapidly changing, traditional (static) predictive models may lose their accuracy. We show that in 7041 patients of whom 534 (7.6%) required invasive mechanical ventilation or died within 14 days that rolling logistic regression models improved both discrimination and the areas under the precision‐recall curves—all *p* < 0.001.

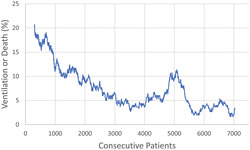

**Abbreviations**
COVID‐19: Severe Coronavirus disease 2019
IMV: invasive mechanical ventilation

## 1 INTRODUCTION

Severe Coronavirus disease 2019 (COVID‐19) can lead to progressive respiratory failure invasive mechanical ventilation (IMV), which ultimately may progress to death. Since its description in Wuhan, China, where treatment was mostly supportive, therapeutic and preventive measures have evolved, including vaccination, corticosteroids (dexamethasone and hydrocortisone), remdesivir and other antiviral agents, and monoclonal antibodies targeting viral proteins.^1^, ^2^, ^3^ After initial concerns that high‐flow nasal oxygen and noninvasive ventilation might cause aerosolization of viruses and COVID infections in healthcare workers proved unfounded, their use has become frequent and may have decreased the need for IMV but, as some studies suggest, may have increased mortality.^4^ Additionally, the virus has undergone frequent mutations affecting the severity of the infection and the ability of anti‐COVID therapies to prevent severe disease.^5^ Case fatality rates and the need for IMV have varied greatly over time and between different strains.^6^, ^7^, ^8^, ^9^

Predicting IMV or mortality can allow improved resource utilization, such as transferring patients to a more intensive level of care, patient and family discussions regarding goals of care, and identifying potential subjects for prospective studies. However, model usefulness, among other factors, depends on predictive ability. Although models may have been externally validated, they may still lose predictive ability as the disease presentation or severity changes or new therapies mitigate its severity. If the predictive ability of the model changes with time, the models may need to be recalibrated or redeveloped to maintain predictive utility. A variety of models have attempted to predict which patients are at risk for clinical decompensation^10^, ^11^; however, these techniques may be limited by rapid evolution in the clinical course of COVID‐19. Models and analytical techniques equipped to dynamically change with the course of COVID‐19 are currently lacking.

The primary purpose of this study is to determine if the predictive ability of a statistical model can be improved through using rolling logistic regression rather than a static logistic regression model and secondarily to determine if the strength of individual predictors changes over time.

## 2 METHODS

This study was approved by the Institutional Review Board approval (University of Michigan HUM00181493), which waived informed consent. All items from the Strengthening the Reporting of Observational Studies in Epidemiology (STROBE) checklist were followed. Patients were included if they were admitted to any of the five Henry Ford Medical Centers (Main, Macomb, West Bloomfield, Wyandotte and Allegiance) between 22 March 2020 and 18 May 2021 or University of Michigan Medical Center between 4 March 2020 and 17 July 2021 and were at least 18 years old on admission. Patients were excluded if they were intubated or died within 4 h of arrival hospital. Both centres serve as primary hospitals for their local populations and as tertiary referral centres. Data from the Henry Ford system were extracted from the electronic health records by a programmer. The individual hospital of each Henry Ford patient was not identified. University of Michigan data were extracted using DataDirect (Ann Arbor, MI). All data were then combined into one dataset for all analyses. We obtained demographics (age, sex and race), vital signs (heart rate, blood pressure, respiratory rate, temperature and pulse oximetry) on admission and throughout their hospital stay, laboratory values (white cell count, triglyceride, LDH, D‐dimer, C‐reactive protein, ferritin, high‐sensitivity troponin and urea nitrogen), Elixhauser comorbidities (diabetes mellitus, COPD, hypertension and heart failure), oxygen use and amount and the outcomes of IMV and mortality.

As previously published,^11^ if the FiO_2_ was provided, we included those values in our analysis. If the O_2_ flow rate was provided, we converted it to FiO_2_ by adding 0.038 for each L/min of supplemental oxygen. Venturi masks and high‐flow nasal cannula were recorded in the chart as FiO_2_. Non‐rebreather masks were considered to supply FiO_2_ = 0.70. Even though the actual FiO_2_ for face masks and nasal cannula will vary from person‐to‐person depending on factors such as tidal volume and respiratory rate, we used these conversion factors to be consistent across all patients.^11^, ^12^ We created one calculated variable, S/F = SpO_2_/FiO_2_.^12^

Data were analysed at 4‐h intervals, starting 4 h after arrival. All variables were entered in the models along with the change in the vital sign, oxygenation and laboratory variables across the 4‐h interval. If no new laboratory or vital signs were recorded in the 4‐h interval, the previous values were carried forward, and the 4‐h change in those variables was set equal to zero. If a laboratory value had not been obtained prior to that interval, the value was imputed as the midpoint of the reference range (triglyceride 100 md/dL, LDH 210 U/l, D‐dimer 0.25 mcg/mL, C‐reactive protein 9 mg/dl, ferritin 180 ug/L, high‐sensitivity troponin T 10 ng/l and urea nitrogen 10 mg/dl). In three separate models, the data at each 4‐h point were used to predict IMV or death within (1) the next 4 h, (2) the next 8 h and (3) the next 24 h.

### 2.1 Statistics

Variables are presented as mean ± standard deviation, median and interquartile range or frequency and percentage, discrimination as c‐statistic ± standard error. We first constructed and tested the ability of a model created on an initial cohort of patients with COVID (construction population) to remain accurate by using logistic regression with forward selection to generate a model on the first 300 patients with COVID, then tested that logistic regression model on the subsequent patients (static logistic regression model). We assessed the discrimination of the model as the area under the receiver operator characteristic curve (c‐statistic). As we expected the patient population to be imbalanced (few patients died or received IMV compared to the many who did not), we further assessed the models using precision‐recall curves as these are more informative when the population is imbalanced.^13^ Comparison of c‐statistics was assessed with the method of Hanley and McNeil, 95% confidence intervals of the area under the precision‐recall curves were calculated with the method of Boyd et al. and the statistical significance determined by bootstrapping. *p* < 0.05 denoted statistical significance.^14^, ^15^

Next, we created a rolling model by using a sliding window of patients to create a logistic regression model, then testing that model on the next patient.^16^ The window then slid one patient over to the right (newer patient) and a variable number on the left to keep the number of patients with adverse outcomes constant, equal to the number of adverse outcomes in the initial 300 patients.(Appendix) This sliding process was repeated until all patients had been tested. This allowed the model to continuously evolve as factors associated with IMV or death may have changed. Similar to above, the models were assessed using area under the receiver operator characteristic and precision‐recall curves. Receiver operator characteristic curves plot the true‐positive rate (sensitivity) versus the true‐negative rate (1‐specifity). Precision‐recall curves plot positive predictive value versus sensitivity. They differ from receiver operator characteristic curves by excluding the true‐negative outcomes, which are frequently the most common outcome. All logistic regressions were done using forward stepwise selection with likelihood ratio to reduce the model. *p* = 0.05 for entry and *p* = 0.10 for removal. All statistics were done in SPSS 27 (IBM, Chicago, IL) with *p*‐values <0.05 and 95% confidence intervals that excluded one denoting significance. No adjustments were made for multiple comparisons.

No formal power calculation was done as it would vary based on the number of patients in the window, but a logistic regression of 300 patients with a 20% adverse outcome rate would expect to support six factors for 4‐h prediction.^17^

## 3 RESULTS

**FIGURE 1:**
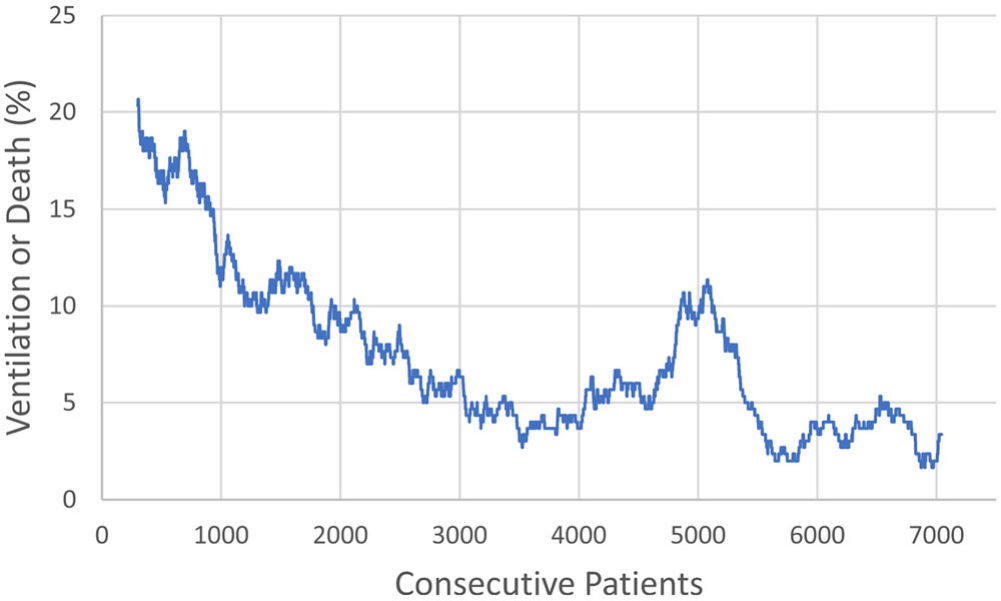
Rolling trends shows the rate of invasive mechanical ventilation or death within 14 days of admission using a rolling rate of 300 consecutive patients. The longest streak without any invasive mechanical ventilation or death was 127 consecutive patients. The longest streak of consecutive patients receiving invasive mechanical ventilation or death was four.

**FIGURE 2:**
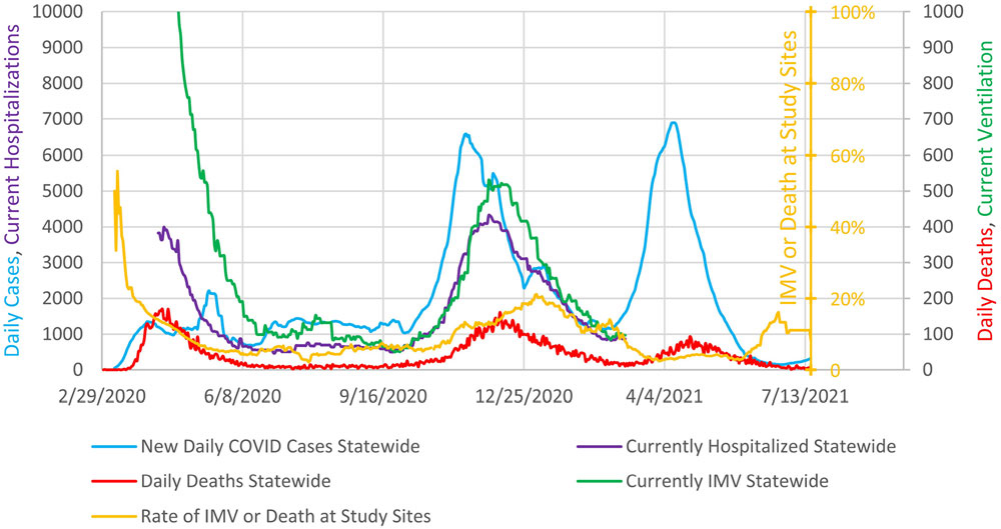
Frequencies of COVID rates and outcomes. Blue line is the number (left axis) of daily COVID‐19 cases in Michigan on a rolling 7 day average as the state did not collect complete data every day. Purple line is the number of COVID‐19 patients in Michigan present in hospital that day (left axis). Red line is the daily number of deaths from COVID in Michigan (right axis). Green line is the number of patients receiving invasive mechanical ventilation in Michigan on that day (right axis). Gold line is the percentage of COVID‐19 patients at the study sites who died or received invasive mechanical ventilation within 14 days of admission. Percentages are calculated over the prior 30 days. Michigan hospitalization and mechanical ventilation numbers are available only from 9 April 2020 to 7 March 2021.

There were 9352 patients admitted with COVID‐19 infection—7484 from the Henry Ford Health System and 1868 from University of Michigan Medical Center. After excluding 2312 patients who received IMV or died on or within 4 h of arrival (many of the patients who received IMV on arrival had been intubated at other hospitals before transfer), the remaining 7041 patients were 51% White, 38% Black and 50% male. They were 62 ± 17 years old. Hypertension was the most common comorbidity. The FiO_2_ values were 0.27 ± 0.15. (Table 1) Of the 7041 patients, 534 (7.6%) received IMV or died within 14 days of arrival. The rate in the initial 300 patients was 20%, then using rolling 300 patient samples, the rate decreased to 3%, before a spike to 11% and then a return to a low rate. (Figure 1) The spike occurred just after the peak of the second statewide surge. However, there was no spike with the third statewide surge. (Figure 2) The models on the initial 300 patients had good discrimination (0.832 ± 0.025 for the 4‐h prediction, 0.806 ± 0.020 for the 8 h and 0.749 ± 0.013 for the 24‐h model) and fair precision‐recall (0.027, 0.045 and 0.073, respectively). However, when these three models (Table 2) were tested on the subsequent 6741 patients, both the discrimination and the area under the precision‐recall curve fell (Table 3).

**TABLE 1:** Admission vital signs, oxygenation, characteristics, laboratory values and comorbidities

Factor	N	Mean	SD
Age (yr)	7041	62	17
Heart rate (min^−1^)	7041	85	17
Mean arterial pressure (mmHg)	7041	91	16
Respiratory rate (min^−1^)	7041	19	6
SpO2 (%)	7041	96	3
Temp (°C)	7041	37.0	0.6
FiO2 (0.01)	7041	0.27	0.15
S/F (%)	7041	409	110

		Median	IQR
C‐reactive protein (mg/dL)	5229	5	4, 11
D‐dimer (mg/dL)	4853	0.3	0.2, 0.5
Ferritin (ng/mL)	5310	258	170, 752
Lactate dehydrogenase (U/L)	4729	258	210, 389
Triglycerides (mg/dL)	3158	65	50, 137
Troponin (pg/mL)	6758	15	0.1, 20
Urea nitrogen (mg/dL)	6860	18	13, 29
White cell count (K/μL)	6836	7.0	5.1, 9.5

		n	%
Male	7041	3540	50
Race	7041		
Black		2659	38
Other/refused/unknown		772	11
White		3610	51
COPD	7041	1267	18
Diabetes mellitus	7041	1285	18
Heart failure	7041	1751	25
Hypertension	7041	5349	76

Abbreviations: IQR, interquartile range; n, number of patients with that characteristic; N, number of patients that had that factor assessed; SD, standard deviation.

**TABLE 2:** (top) Logistic regression associated with need for invasive mechanical ventilation or death within 4 h based on the first 300 patients who were studied for 13, 254 time intervals. Intubation with mechanical ventilation or death occurred in 61 patients (20%) and 61 intervals (0.5%). (middle) Logistic regression associated with need for invasive mechanical ventilation or death within 8 h based on the first 300 patients who were studied for 13 254 time intervals. Intubation with mechanical ventilation or death occurred in 61 patients (20%) and 109 intervals (0.8%). (bottom) Logistic regression associated with need for invasive mechanical ventilation or death within 24 h based on the first 300 patients who were studied for 13,254 time intervals. Intubation with mechanical ventilation or death occurred in 61 patients (20%) and 285 intervals (2.2%).

4‐h model				
Factor	*p*‐Value	Odds ratio	95% LCI	95% UCI
Mean arterial pressure (mmHg)	0.028	1.019	1.002	1.036
Respiratory rate	<0.001	1.063	1.029	1.098
Triglycerides (mg/dL)	0.002	0.994	0.989	0.998
D‐dimer (mg/L)	0.014	1.667	1.108	2.509
Ferritin (100 ng/ml)	0.009	1.011	1.003	1.019
SpO2 (%)	0.011	0.959	0.929	0.990
FiO2 (0.10)	<0.001	1.456	1.327	1.597
Constant	0.001	0.003		

8‐h model				
Factor	*p*‐value	Odds ratio	95% LCI	95% UCI
Mean arterial pressure (mmHg)	0.009	1.017	1.004	1.030
Respiratory rate	<0.001	1.068	1.042	1.095
Triglycerides (mg/dL)	<0.001	0.994	0.991	0.997
D‐dimer (mg/L)	0.004	1.590	1.164	2.172
Ferritin (100 ng/ml)	<0.001	1.013	1.006	1.019
SpO2 (%)	0.009	0.964	0.938	0.991
FiO2 (0.10)	<0.001	1.399	1.304	1.501
Constant	<0.001	0.004		

24‐h model				
Factor	*p*‐Value	Odds ratio	95% LCI	95% UCI
Mean arterial pressure (mmHg)	0.020	1.010	1.002	1.018
Respiratory rate	<0.001	1.050	1.031	1.069
Triglycerides (mg/dL)	<0.001	0.994	0.992	0.996
D‐dimer (mg/L)	<0.001	1.645	1.340	2.019
Ferritin (100 ng/ml)	<0.001	1.014	1.007	1.021
White cell count (K/μL)	<0.001	0.918	0.885	0.952
Urea nitrogen (mg/dL)	0.045	0.993	0.987	1.000
SpO2 (%)	<0.001	0.965	0.947	0.984
FiO2 (0.10)	0.043	1.128	1.004	1.268
S/F (%)	<0.001	0.995	0.993	0.998
Race—Black (ref)	0.017	1		
Other/unknown/refused	0.961	0.987	0.594	1.642
White	0.005	0.641	0.471	0.872
Constant	0.703	0.665		

Abbreviations: LCI, lower confidence interval; UCI, upper confidence interval.

**TABLE 3:** Areas under the receiver operator characteristic (discrimination, c‐statistic) and precision‐recall curve

Models created and tested on construction population				
Model	c‐Statistic	SE	PR curve	95% CI
4 h	0.832	0.025	0.027	0.024, 0.030
8 h	0.806	0.020	0.045	0.043, 0.050
24 h	0.749	0.013	0.073	0.069, 0.076

Static models tested on subsequent population				
Model	c‐Statistic	SE	PR curve	95% CI
4 h	0.827^*^	0.011	0.006^****^	0.005, 0.007
8 h	0.794^**^	0.012	0.010^*****^	0.008, 0.012
24 h	0.735^***^	0.012	0.021^******^	0.019, 0.024

Rolling models tested on subsequent population				
Incremental	c‐statistic	SE	PR curve	95% CI
4 h	0.865^*^	0.010	.030^****^	0.027, 0.033
8 h	0.856^**^	0.007	.046^*****^	0.043, 0.050
24 h	0.843^***^	0.005	.076^******^	0.071, 0.080

Abbreviations: 95% CI, 95% confidence interval; PR curve, area under the precision‐recall curves; SE, standard error of the c‐statistic.

^*^ *p* < 0.001.

^**^ *p* < 0.001.

^***^ *p* < 0.001.

^****^ *p* < 0.001.

^*****^ *p* < 0.001.

^******^ *p* < 0.001.

**FIGURE 3:**
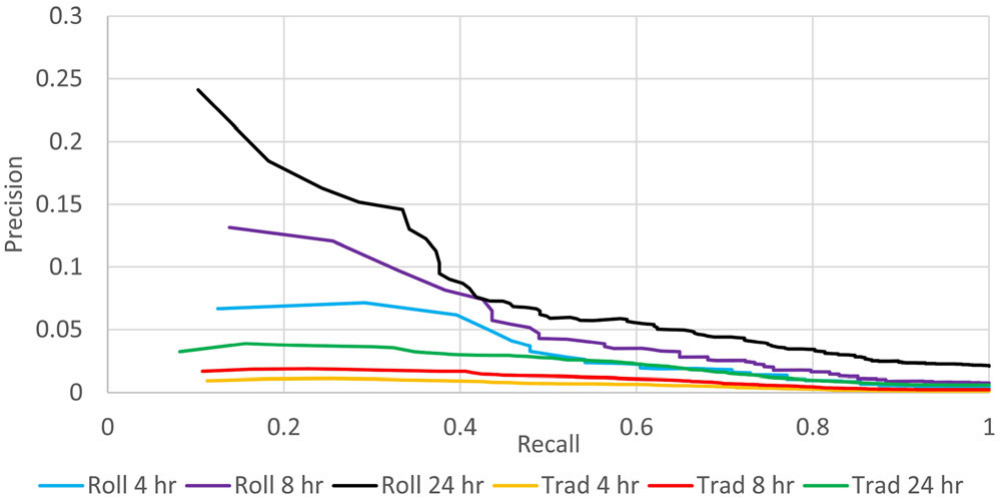
Precision‐recall curves for the rolling (roll) and static (Trad) logistic regression models. The number needed to identify is calculated as 1/precision at any recall value. For example, the number needed to identify one patient who will receive mechanical ventilation or die at recall = 0.2 is 5.6, 7.9 and 15 for the rolling 24‐, 8‐ and 4‐h models and 27, 56 and 83 for the static 24‐, 8‐ and 4‐h models, respectively. At a recall = 0.8, the numbers needed to identify are 29, 57 and 100 for the rolling 24‐, 8‐ and 4‐h models and 100, 250 and 333 for the static 24‐, 8‐ and 4‐h models, respectively.

Using the rolling logistic regressions to continuously update the models, we found improved discrimination: 0.865 ± 0.010, 0.856 ± 0.007 and 0.843 ± 0.005 for the 4‐, 8‐ and 24‐h models, respectively; all *p* < 0.001 compared to the static logistic regressions. (Table 3) Similarly, the areas under the precision‐recall curves improved from 0.006, 0.010 and 0.021 with the static models to 0.030, 0.046 and 0.076 for the 4‐, 8‐ and 24‐h rolling models, respectively, all *p* < 0.001 (Figure 3).

**FIGURE 4:**
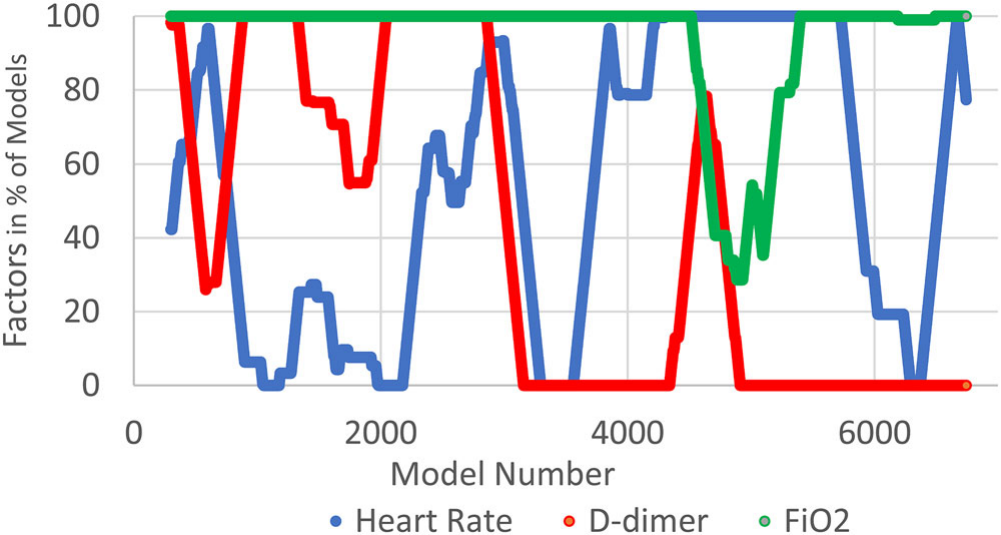
Plot showing the percent of times FiO2, heart rate and D‐dimer are statistically significant factors in 300 consecutive rolling regressions associated with invasive mechanical ventilation or death within the next 4 h. Plot shows that FiO2 was in all the models until model #4515. Close to simultaneously, D‐dimer percentage in the models has increased to 100% but falls before FiO_2_ starts to increase back to 100%. It's place in the models is taken by several other less frequently statistically significant factors (not shown for clarity).

FiO_2_, present in 94% of the rolling regression models, and respiratory rate (88%) were the most common factors in the rolling regressions associated with mechanical ventilation or death within 4 and 8 h (FiO_2_ 92% and respiratory rate 83%). For the 24‐h model, whereas FiO_2_ remained the most common factor (76%), the frequency of respiratory rate in the models had fallen to 24% and temperature (57%) became the second most common factor. (Table 4) C‐reactive protein and D‐dimer were the most common laboratory values in the models. Changes in vital signs or in laboratory values were infrequent factors in the rolling models. (Table 4) Comorbidities were factors in a moderate number of models, whereas age and sex were rare, and race presents only in the construction model, not in any of the subsequent rolling models. The presence of even common factors was not consistent but varied with time. Figure 4 shows how the three most common factors varied with time. In particular, FiO_2_ was not in the 4‐h model when the spike in mechanical ventilation or death occurred but was otherwise present.

**TABLE 4:** Count of number of times each factor is in a rolling regression model. Δ—Change in factor value from the previous 4‐h value. S/F ‐SpO_2_/FiO_2_

	4 h		8 h		24 h	
Factor	*N*	%	*N*	%	*N*	%
Age	52	1%	191	3%	71	1%
COPD	1732	26%	1577	23%	1240	18%
C‐reactive protein	3288	49%	3321	49%	2402	36%
D‐dimer	2663	40%	2477	37%	1047	16%
Diabetes mellitus	1587	24%	1503	22%	1205	18%
Ferritin	338	5%	409	6%	314	5%
FiO2	6361	94%	6184	92%	5119	76%
Heart failure	972	14%	1086	16%	849	13%
Heart rate	3652	54%	1558	23%	930	14%
Hypertension	960	14%	934	14%	643	10%
Lactate dehydrogenase	1134	17%	1046	16%	1492	22%
Mean arterial pressure	1286	19%	948	14%	854	13%
Respiratory rate	5903	88%	5593	83%	1614	24%
S/F	662	10%			166	2%
Sex	17	0.3%	215	3%	305	5%
SpO_2_	2570	38%	2029	30%	1493	22%
Temperature	3223	48%	4414	65%	3873	57%
Triglycerides	564	8%	682	10%	452	7%
Troponin	1102	16%	1088	16%	1103	16%
Urea nitrogen	1156	17%	1091	16%	508	8%
White cell count	368	5%	363	5%	382	6%
ΔCRP					1550	23%
Δheart rate	695	10%	131	2%		
Δmean arterial pressure	714	11%	63	1%	874	13%
Δrespiratory rate	891	13%	937	14%	399	6%
ΔS/F	683	10%	1077	16%		
ΔSpO_2_	958	14%	318	5%	128	2%
Δtemperature					246	4%
Δtriglycerides	134	2%	23	0.3%		
Δtroponin					389	6%

## 4 DISCUSSION

We found that use of the rolling regression models by continuously updating the data included in the models (excluding older and adding the most recent patient) improved the models during a time when the disease, treatment and outcome were rapidly changing. Unlike the static regression models, the rolling logistic regression models maintained their discrimination and precision‐recall values close to the values in the construction population. Our finding that after a period of improved outcomes, the rate of IMV and death spiked up before decreasing again is similar to a study from the United Kingdom that showed a similar decrease followed by an increase in mortality, which the authors attributed to the impact of the B117 variant.^18^ As we do not have genetic sequencing data, we are limited in not knowing if our sudden spike in adverse outcomes is related to a COVID variant or to other reasons.

Logistic regression models are frequently judged by their ability to discriminate between the two outcomes. However, the c‐statistic (area under the receiver operator characteristic curve) may not be a good metric when one of the two outcomes is uncommon. Precision‐recall curves, which exclude true negatives from the calculation, may be a better metric of the models' utility.^13^ Precision‐recall curves also make it easy to calculate the number needed to identify. (Figure 3) Identifying patients at high risk for IMV or death may improve outcomes by earlier and more intensive treatment. It also identifies a group of patients for enrollment in prospective studies by, given their higher likelihood of IMV or death, improving power and decreasing the number of patients needed for the study.

We found that most factors were at least occasionally associated with IMV and death. However, a few factors were frequently included in the models. In particular, FiO_2_ appeared in most models. Study is needed to determine why FiO_2_ lost its predictive utility during the spike to 11% rate of IMV or death, whether it relates to changes in disease phenotype, treatment or is merely a result of random fluctuation. FiO_2_ was initially replaced by D‐dimer in the models, coincident to the December 2020 surge with its higher rate of IMV or death.

SpO_2_, a measure of oxygenation, and S/F, appeared infrequently in the models, which differs from previous studies that found S/F to be highly associated with the need for IMV or death; however, these studies did not separately analyse FiO_2_.^11^, ^19^, ^20^, ^21^ A rising FiO_2_ should be taken as one of the warning signs for impending death or need for IMV. Vital signs, particularly, respiratory rate and temperature, were also commonly present in the models. Abnormal vital signs are components of Systemic Inflammatory Response Syndrome, Modified Early Warning and quick Sequential Organ Failure Assessment screens for impending deterioration.^22^, ^23^, ^24^, ^25^ The 24‐h models had temperature as a frequent predictor, but in the 8‐ and 4‐h models, it had become less common, and respiratory rate had become much more common. This suggests that temperature may be an earlier warning sign (occurring at 24 h), whereas respiratory rate becomes a predictor of more imminent deterioration (8 h).

Although some previous studies have found age, sex and race to be factors associated with worse outcomes in COVID infection,^26^, ^27^, ^28^ we found these factors to be rarely associated with IMV and death. Our study differs from these by the inclusion of different factors. Ho et al. in a population‐based study found older age to be markedly associated with increased mortality.^26^ Our study differs by only including hospital patients. Many older persons with comorbidities infected with COVID were not hospitalized but instead died in nursing homes and extended care facilities.^29^ Notably, Nguyen et al., who found an excess of males receiving IMV or dying in the Vizient database of >300 000 patients at >650 academic medical centres, included only administrative data and not vital signs and laboratory data.^27^ Males and females may present with different vital signs and laboratory values, which may be more closely associated with outcomes. Our study found that after adjusting for confounders, age and sex had little effect on IMV or death, perhaps related to studying only hospitalized patients and by including vital signs and laboratory values, which may have acted as mediators between age and sex and the adverse outcomes. Although initial population‐based studies found higher death rates among Black than White American, CDC data had suggested that by October 2020, the rates had reversed, with White Americans now having a higher rate.^28^ Our study is similar to this in finding an initially higher adjusted mortality in Black than White patients, which then quickly disappeared. However, we did not find a higher mortality in White patients.

Rolling regressions can easily be integrated with the electronic health record to continuously update and provide clinicians with the best, most current prediction models. As vulnerable populations, disease characteristics and treatments all change, the models will evolve to stay concurrent; however, further study is needed in different populations including ones where the disease is relatively invariant.

There are several limitations to this study. First, this is only a six‐hospital study from the same geographic area (southeastern Michigan). Studies from other geographic areas or with different healthcare systems may not only find different factors associated with IMV or death but find different discrimination and precision‐recall values of their models. We were also limited in being provided only a few comorbidity and laboratory values for analysis. Inclusion of more comorbidities and more laboratory values might have improved the models. Despite this, our limited data collection produced good discrimination and fair precision‐recall values. Third, patients had missing laboratory values and vital signs—laboratory tests were not ordered and vital signs may not have been obtained every 4 h. Tests and vital signs tend to be ordered and obtained based on clinical course and need. Rather than imputing missing values, we carried forward the most recent value or if a laboratory test had not been obtained, we assigned it a normal value, similarly to APACHE III.^30^ The utility of models developed by institutions is partially dependent on how frequently vital signs and laboratory tests are obtained, but how often data need to be collected to maximize utility of rolling logistic regression models remains to be understood. We did not include the patient's hospital in the analyses. This might bias the analysis in unknown ways. Finally, we are limited by not knowing vaccination status and treatments. Use of steroids, monoclonal antibodies, antiviral agents and varying modalities of respiratory therapy, such as prone position, heated high‐flow nasal cannula and noninvasive mechanical ventilation, were not available to us. Including these potential therapies in the models would allow us to assess their efficacy, and inclusion with interaction terms would allow us to determine if their efficacy was related to other conditions, such as with FiO_2_.

One of the strengths of this study is the use of precision‐recall curves to display utility. Although receiver operator characteristic curves and discrimination are frequently used, by ‘fattening up’ on easy to identify true‐negative patients, despite the high c‐statistic value, they may not be useful when the adverse event rate is low.^13^ Precision‐recall curves better characterize the utility of the model and allow for easy determination of the number needed to identify to find one patient who will develop the adverse outcome (Figure 3).

In conclusion, we found that rolling logistic regressions to maintain a more contemporaneous model performed better than did the static logistic regression using older data when tested on subsequent patients. We also found that increasing FiO_2_ and abnormal vital signs were the factors most commonly associated with IMV and mortality.

## CONFLICT OF INTEREST

Dr. Nicholas Douville received support from a Foundation for Anesthesia Education and Research (FAER)—Mentored Research Training Grant (MRTG). None of the other authors has any disclosures. This research was supported solely by university and departmental sources.

## ETHICS STATEMENT

This study was approved by the Institutional Review Board (University of Michigan HUM00181493), which waived informed consent and permitted publication.

## AUTHOR CONTRIBUTIONS

**Milo Engoren:** conceptualization, methodology, software, validation, formal analysis, investigation, data curation, writing—original draft, visualization, project administration.

**Carlo Pancaro:** conceptualization, investigation, writing—review and editing.

**Nicholas S. Yeldo:** investigation, resources, writing—review and editing, project administration.

**Lotfi S. Kerzabi:** software, investigation, resources, data curation, writing—review and editing.

**Nicholas Douville:** conceptualization, investigation, writing—review and editing, visualization.

## Data Availability

The data that support the findings of this study are available from University of Michigan and Henry Ford Medical Center upon request and approval.

## APPENDIX A

$$\left\{N_{1,}\;N_{2},N_{3},\ldots \;N_{299},N_{300}\right\},\;N_{301},\;N_{302},\;\ldots \;\text{where}\;N_{1}=0,\;N_{2}=1,\;N_{3}=0,\;\ldots$$

where 1 indicates received mechanical ventilation or died in the next time interval and 0 indicates the opposite for each patient N_i_.

The logistic regression is first constructed on patients N_1_–N_300_ and tested on N_301_. The window then slides one patient to the right (N_301_) and patients N_1_ and N_2_ are dropped to keep the number of patients with the outcome of mechanical ventilation or death fixed at a constant number (*n* = 60). A new logistic regression model is then constructed using patients N_3_–N_301_ and tested on N_302_. The process is repeated until N_last_ is tested. The total numbers of correct and incorrect predictions are counted and used to calculate discrimination and the area under precision‐recall curves.
